# New Operation for the Radical Cure of Varicocele

**Published:** 1849-01

**Authors:** S. D. Gross

**Affiliations:** Professor of Surgery in the Medical Department of the University of Louisville, Louisville, Ky.


### ARTICLE XI.

*New Operation for the Radical Cure of Varicocele.* By S. D. Gross, M. D., Professor of Surgery in the Medical Department of the University of Louisville.

The following operation, for the radical cure of varicocele, I have performed eight times within the last few years. The patients were all young men of good constitution, and they all recovered without a single bad symptom. The cure, so far as I have been able to learn, promises to be permanent in every instance.

During the operation, the patient may lie down, sit in a chair, or stand up, as may be most convenient. The scrotum, previously divested of hair, is rendered tense by grasping it behind with the left hand. A vertical incision, scarcely an inch in length, is made over the anterior part of the tumor, dowd to the enlarged veins, which are next carefully isolated from the accompanying duct, artery, and nerves, by a few touches with the point of the scalpel. This constitutes the first step of the operation. The second consists in passing a short, thick sewing needle—a No. 1 of the milliner, underneath two or three of the larger trunks, and winding around it a stout thread, either elliptically, or in the form of the figure 8. The ligature is drawn with great firmness, so as to indent the coats of the vessels, and put an immediate stop to the circulation. The operation is finished by closing the wound carefully with one or two sutures, or a few strips of court-plaster. The patient is now put to bed, the scrotum is supported with a silk handkerchief, and light diet is enjoined, At the end of twenty-four, or, at the most, thirty-six hours, the blood in the constricted veins is sufficiently coagulated to justify their division, and the removal of the needle. This is readily effected by insinuating a narrow, sharp-pointed bistoury underneath the vessels, with its back towards the needle.

Should symptoms of inflammation arise after the operation, or, in other words, should the parts become red, tender, and swollen, recourse must be had to antiphlogistics, and to the application of cold water, or solutions of acetate of lead and opium. The patient may usually sit up in five or six days, and in a few more he may be permitted to walk about. The little wound soon cicatrizes; and the induration, caused by the coagulation of the blood between the testis and the seat of the constriction, gradually disappears by absorption. The period required for this rarely exceeds a month.

The advantages of the above operation are, first, its perfect simplicity and the facility with which it may be executed; secondly, its freedom from pain and hemorrhage; thirdly, the certainty with which we may avoid injury to the spermatic artery, duct, and nerves; fourthly, the little inconvenience or suffering which the patient experiences after it has been performed; and fifthly, the rapidity of the cure. These considerations will, I think, be found sufficient to recommend this method to the favorable notice of practitioners. Most of the operations described in the books are complicated, severe, and dangerous.

It occasionally happens, in this affection, that the scrotum is very flabby and pendulous. When this is the case, the cure will hardly be complete unless the surgeon retrenches the redundant structures. I have been obliged to resort to this expedient only once in my operations. A portion of scrotum, nearly of the size of a large hand, was excised with the scalpel, and the wound closed by the continued suture, which I consider far preferable, under such circumstances, to the interrupted or twisted.

Louisville, Ky., July, 1848. [*Am. Jour. Med. Sci.*

